# A theory-based multi-component intervention to increase reactive balance measurement by physiotherapists in three rehabilitation hospitals: an uncontrolled single group study

**DOI:** 10.1186/s12913-018-3533-8

**Published:** 2018-09-19

**Authors:** Kathryn M. Sibley, Danielle C. Bentley, Nancy M. Salbach, Paula Gardner, Mandy McGlynn, Sachi O’Hoski, Jennifer Shaffer, Paula Shing, Sara McEwen, Marla K. Beauchamp, Saima Hossain, Sharon E. Straus, Susan B. Jaglal

**Affiliations:** 1 Department of Community Health Sciences, University of Manitoba, 379–753 McDermot Avenue, Winnipeg, MB R3E 0W3 Canada; 2 Centre for Healthcare Innovation, 753 McDermot Avenue, Winnipeg, R3E 0W3 MB Canada; 3 Toronto Rehabilitation Institute- University Health Network, 550 University Avenue, Toronto, ON M5G 2A2 Canada; 4 Faculty of Medicine, University of Toronto, 1180-1 King’s College Circle, Toronto, ON M5S 1A8 Canada; 5 Department of Physical Therapy, University of Toronto, 160–500 University Avenue, Toronto, ON M5G 1V7 Canada; 6 Department of Health Sciences, Brock University, 1812 Sir Isaac Brock Way, St. Catharines, L2S 3A1 ON Canada; 7 West Park Healthcare Centre, Toronto, Canada; 8 School of Rehabilitation Science, McMaster University, 1400 Main Sreet West, Hamilton, ON L8S 1C7 Canada; 9 Sunnybrook Health Sciences Centre – St. John’s Rehab, 285 Cummer Avenue, Toronto, ON M2M 2G1 Canada; 10 Bridgepoint Active Healthcare – Sinai Health System, 1 Bridgepoint Drive, Toronto, ON M4M 2B5 Canada; 11 Li Ka Shing Knowledge Institute – St. Michael’s Hospital, 30 Bond Street, Shuter 2–026, Toronto, ON M5B 1W8 Canada; 12 Department of Geriatric Medicine, University of Toronto, Toronto, Canada

**Keywords:** Falls prevention, Postural balance, Evidence based practice, Implementation science, Health research

## Abstract

**Background:**

Most implementation interventions in rehabilitation, including physiotherapy, have used passive, non-theoretical approaches without demonstrated effectiveness. The goal of this study was to improve an important domain of physiotherapy practice – reactive balance measurement – with a targeted theory-based multi-component intervention developed using the Theoretical Domains Framework. The primary objective was to determine documented reactive balance measure use in a 12-month baseline, during, and for three months post- intervention.

**Methods:**

An uncontrolled before-and-after study was completed with physiotherapists at three urban adult rehabilitation hospitals in Ontario, Canada. The 12-month intervention included group meetings, local champions, and health record modifications for a validated reactive balance measure. The primary outcome was the proportion of records with a documented reactive balance measure when balance was assessed pre-, during- and post-intervention. Secondary outcomes were changes in use, knowledge, and confidence post-intervention, differences across sites, and intervention satisfaction.

**Results:**

Reactive balance was not measured in any of 211 eligible pre-intervention records. Thirty-three physiotherapists enrolled and 28 completed the study. Reactive balance was measured in 31% of 300 eligible records during-intervention, and in 19% of 90 eligible records post-intervention (*p* < 0.04). Knowledge and confidence significantly increased post-intervention (all *p* < 0.05). There were significant site differences in use during- and post-intervention (all *p* < 0.05). Most participants reported satisfaction with intervention content (71%) and delivery (68%).

**Conclusions:**

Reactive balance measurement was greater among participants during-intervention relative to the baseline, and use was partially sustained post-intervention. Continued study of intervention influences on clinical reasoning and exploration of site differences is warranted.

**Electronic supplementary material:**

The online version of this article (10.1186/s12913-018-3533-8) contains supplementary material, which is available to authorized users.

## Background

Rehabilitation, a specialized component of health care that involves interventions to optimize function and reduce disability in individuals with health conditions in interaction with their environment [1], represents a distinct implementation research context. Rehabilitation interventions are often complex [2], and rehabilitation is practiced by multiple professions including (but not limited to) physiotherapists, occupational therapists, speech-language pathologists, and physical and rehabilitation medicine physicians [3]. In Canada, physiotherapy is the largest non-medicine rehabilitation profession [4], with over 21,000 individuals employed as physiotherapists in 2016 [5].

There is a dearth of implementation research among rehabilitation clinicians, including physiotherapists [6, 7]. For example, the most recent systematic review of physiotherapy, occupational therapy, and speech language pathology knowledge translation strategies included just 26 studies and reported no clear delineation of the effect of different strategies on any outcome [7]. Most interventions adopted primarily passive education strategies, although two physiotherapy studies that reported consistent positive effects used active multi-component approaches. Most rehabilitation and physiotherapy implementation interventions to date have not been developed using behavioral theory [7–9]. The absence of theory-based implementation interventions for rehabilitation clinicians is noteworthy as the use of theory is postulated to increase the generalizability of implementation interventions and facilitate investigation of causal mechanisms between intervention components and outcomes [10].

Ongoing gaps in rehabilitation implementation research present an important opportunity to develop and test theory-based, multi-component interventions. The goal of this study was to improve practice in a key rehabilitation discipline with a theory-based multi-component intervention. The focus for improved practice was a critical functional skill of relevance to physiotherapy practice: reactive balance. Reactive balance, the ability to recover from postural instability through a rapid corrective postural muscle response, step or grasp [11], is a fundamental skill for avoiding falls [12]. Falls represent a major health concern for older adults and many clinical populations with neuromuscular and/ or musculoskeletal impairment due to their high frequency [13], potential for serious injury [14] and high health care costs [15]. Multiple studies have identified reactive balance as an independent risk factor for falls, and systematic reviews from multiple populations have demonstrated that targeted reactive balance exercise training with external perturbations significantly reduced the number of falls and individuals falling [16–20].

Although treatment of balance impairments is a recognized key component of physiotherapy practice, reactive balance is not explicitly targeted by physiotherapists. Multiple surveys of Canadian balance assessment practices have found that less than half of participating physiotherapists reported regularly assessing reactive balance when treating adults with balance impairment [21, 22]. One retrospective study of balance assessment among adults aged 65 years and older who were admitted to a Canadian rehabilitation hospital in 2009–10 determined that just 2% of randomly-selected records included an explicit assessment of reactive balance [23]. One study exploring factors influencing reactive balance measurement identified multiple barriers. These included perceptions of lack of knowledge, lack of time, lack of access to and unavailability of reactive balance measurement tools, and inappropriateness of reactive balance measurement for specific populations. This study also identified a key facilitator: more than 80% of participating physiotherapists reported wanting to improve their assessment of reactive balance [24].

The overall aim of this study was to improve physiotherapist measurement of reactive balance in a rehabilitation setting. The primary objectives were to determine (i) documented use of a standardized reactive balance measure in people receiving a balance assessment during a baseline period of routine care, (ii) documented use of a standardized reactive balance measure by physiotherapists during participation in a theory-based, multi-component intervention developed to increase reactive balance measurement; and (iii) documented use of a standardized reactive balance measure for 3 months following the intervention. The secondary objectives of the study were to determine variations in reactive balance measure use across three sites, to identify changes in participating physiotherapists’ reactive balance knowledge and measurement confidence before and after the intervention, to determine satisfaction with the intervention, and to compare changes in knowledge and confidence and satisfaction across sites.

## Methods

### Study design and conceptual foundations

A quasi-experimental, multi-site, uncontrolled study that spanned a 27-month time frame was completed. The application of the Knowledge-to-Action Framework [25] as a guiding framework for this research program has been previously reported [26]. With this framework, we previously completed the *identify problem* and *assess barriers to knowledge use* phases [21, 23, 24, 27]. The present study addressed the phases of *adapting to local context; selecting, tailoring and implementing interventions; monitoring use; evaluating outcomes* and *sustaining knowledge use*. Institutional approval including research ethics approval was obtained at all sites.

### Setting

The study was conducted at three urban adult rehabilitation hospitals in Ontario, Canada. All sites provide inpatient and outpatient rehabilitation care for multiple clinical populations with balance impairment, including older adults (aged 65 years and older) and people living with neurological disorders, musculoskeletal impairments, trauma, amputation, and general deconditioning.

### Eligibility and recruitment

Physiotherapists working in relevant clinical programs at the study sites who were not already involved in ongoing clinical balance research were eligible to participate in the study. There were 67 eligible physiotherapist full-time equivalent (FTE) positions at the time of the study, representing 75 individual physiotherapists (64 full-time and 11 part-time). Eligible physiotherapists were identified by physiotherapist research team members (onsite local champions- described below), and were actively recruited by a research coordinator who introduced the study at each site during a clinical meeting, then individually invited all eligible physiotherapists via email.

### Theory-based multi-component intervention

A 12-month intervention was administered at each site. Details of intervention development have been reported elsewhere [28] and are described briefly here. Previously-identified factors influencing reactive balance assessment informed the selection of the reactive balance measure [29] and were mapped to the Theoretical Domains Framework (TDF) [30, 31]. The intervention (Table 1) targeted eight TDF domains through eight established behavior change techniques [32] that were incorporated into three intervention components that were supported by existing behaviour change and implementation evidence: (i) onsite local champions who were physiotherapist research team members with an advanced practice or management role at each site and were identified at study conception through networking and were involved in all aspects of study planning, intervention delivery, data collection and analysis, (ii) seven group meetings that included didactic education, hands-on practice, and ongoing discussion, and (iii) health record modifications that included development of a reactive balance measure administration form customized for the study that included instructions and scoring guidance, and met institutional specifications (Additional file 1).

**Table 1:** Intervention foundation [28]

Theoretical Domains Framework domain	Effective technique for changing domain	Intervention component targeting domain
Knowledge	Information regarding behavior, outcome	Group meeting
Skills	Rehearsal of relevant skills	Group meeting, local champion
Social/ professional role and identity	Feedback	Group meeting, local champion
Beliefs about capabilities	Social process of encouragement, pressure, support	Local champion
Beliefs about consequences	Problem-solving, decision-making, goal-setting	Group meeting
Intentions	Modeling/demonstration of behavior by others	Group meeting, local champion
Memory, attention, and decision processes	Environmental changes	Health record modification
Environmental context	Prompts, triggers, cues	Health record modification

With regards to the intervention components, the local champions worked actively to engage participants and foster adoption of the reactive balance measure through a variety of activities as needed throughout the study [28]. These included scheduling and coordinating group meetings, communicating study goals and objectives, co-facilitating group meeting discussions to tailor activities to each site, modeling use of the reactive balance measure in their caseload and encouraging use among participants, providing ongoing mentorship and problem solving with participants between meetings, answering questions and brokering interactions between participants and the larger offsite research team, as well as co-developing and customizing the reactive measure administration forms, and assisting with study administrative coordination as needed. The group meetings included one 60-min didactic education session, one 60-min hands-on practice session, and five 60-min bi-monthly check-in discussions. The didactic education session was delivered by the principal investigator and included foundational content on reactive balance, its measurement, and the reactive balance measure used in the study. The hands-on practice session was led by two physiotherapists who were regular users of the measure and included viewing of training administration and scoring videos and test practice among participants. The bi-monthly check-in sessions included informal discussion of questions and concerns about administration and scoring issues, experiences using the test, trouble shooting, etc. Bi-monthly check-in discussions were co-facilitated by the principal investigator, local champion, physiotherapist hands-on practice trainers and the research coordinator. Additional resources, such as test scoring interpretation guidance and visual handouts to use with patients when administering the test, were developed and distributed in an iterative fashion in response to participant requests to the study team. Sample agendas for all three meeting types are included in Additional file 2.

**Fig. 1:**
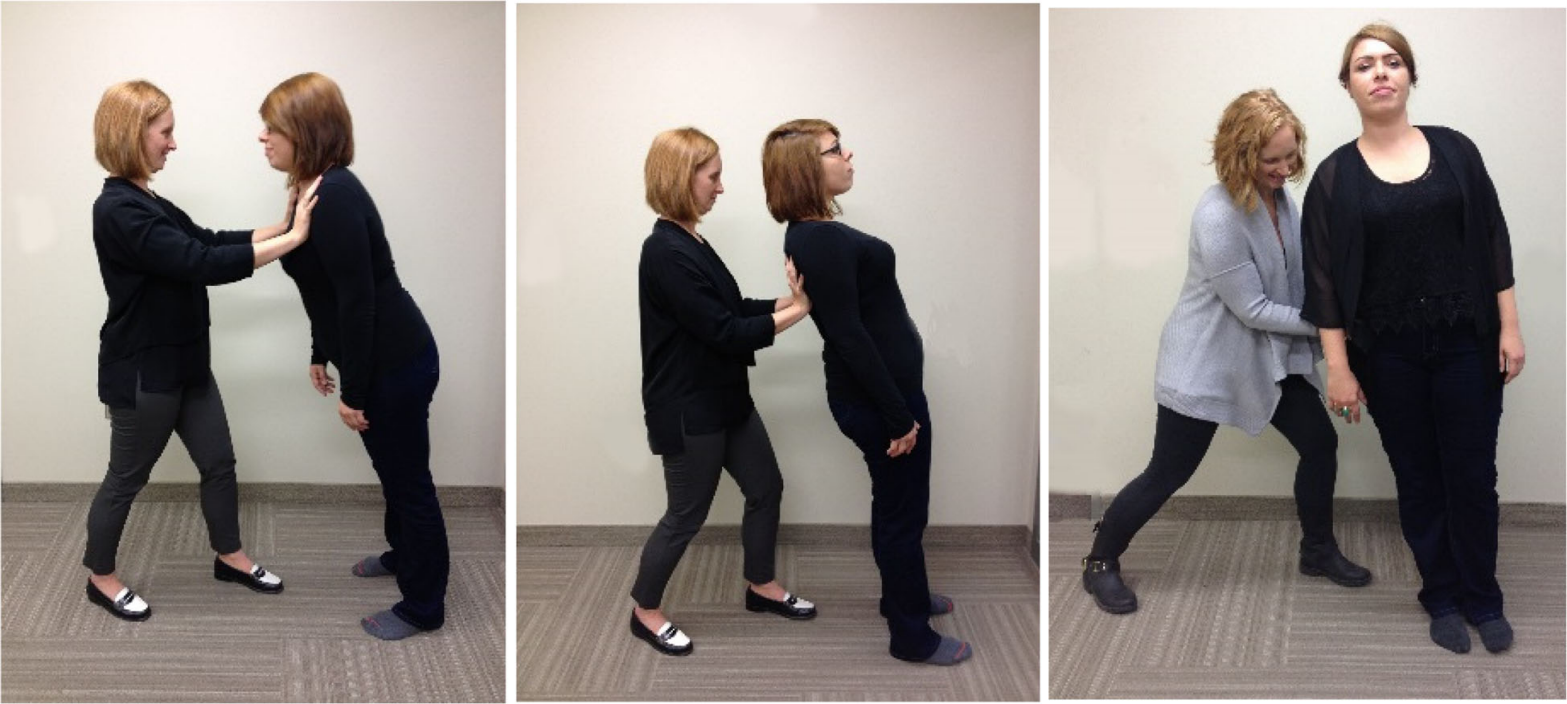
Sample reactive balance measure (postural responses section of the Balance Evaluation Systems Test) administration positions. Administration and scoring guidance videos are available at www.bestest.us

The reactive balance measure selected was the Postural Responses section of the Balance Evaluation Systems Test [29] (Fig. 1). The Balance Evaluation Systems Test is a comprehensive balance measure with established interrater reliability [33], normative data [34], and documented use in multiple clinical populations [35–37]. Each section of the comprehensive measure has been individually tested, and developers have indicated that sections can be used independently [33]. The Postural Responses section is a 6-item measure of reactive balance requiring no equipment. Interrater reliability for this section was found to be excellent (intraclass correlation coefficient = 0.92, 95% confidence interval = 0.85–0.97). This measure was selected because it addressed multiple identified factors influencing reactive balance measurement including availability of tools, appropriate for multiple populations, and (lack of) time [29].

### Data collection

Multiple data collection methods were used to address each objective and were tailored to align with site-specific conditions and data availability.

#### Extraction of Health Record Information

To establish baseline reactive balance measurement practices and estimate reactive balance measure use in patients receiving a balance assessment (primary objective i), 300 randomly-identified health records from the 12-month period pre-intervention were sampled from clinical programs where eligible physiotherapists were working (100 per site). Health records were eligible if they included evidence of balance assessment, either through documented scores from a standardized balance measure or included notation of an informal balance assessment. Pre-intervention health record extraction was not linked to the treating physiotherapist.

To estimate reactive balance measure use during the intervention and for 3 months post- intervention (primary objectives ii and iii), 300 randomly-identified during-intervention and 90 post-intervention eligible health records (with evidence of formal or informal balance assessment) from physiotherapists participating in the study were consecutively reviewed.

Health record data were extracted using a customized data abstraction tool that was adapted from a published chart review of balance assessment practice [23]. Extracted variables included use of the reactive balance measure at any point during treatment (binary – yes/ no) and associated scores, use of other standardized balance measures (binary) and associated scores, and patient characteristics. One team member was involved in data collection at all sites and time points (SH) and trained additional reviewers to ensure consistency of data extraction across reviewers.

#### Questionnaire

To identify changes in physiotherapists’ reactive balance knowledge and measurement confidence before and after the intervention (secondary objective), a customized questionnaire was administered pre- and post-intervention (Additional file 3). The questionnaire was developed by the Principal Investigator in collaboration with the research team and reviewed for face validity and clarity with the onsite local champions. Items addressed self-reported knowledge of reactive balance (ability to define, relation to fall risk, awareness of standardized measures for assessing it; multiple choice questions with “check all that apply” answer options), and self-perceived confidence to measure reactive balance (identify appropriate patients, select appropriate method, safely administer, interpret; 11-point Likert scale). Knowledge and confidence sections were each summed and expressed as total percentage.

To determine participant satisfaction with the intervention (secondary objective), the post-intervention questionnaire also explored satisfaction with the content and delivery of the intervention, intent to continue using the reactive balance measure on completion of the study, and utility of the individual intervention components (7-point Likert scale). Satisfaction ratings were collapsed into two categories: “positive” (combining positive and strongly positive categories) and “not positive” due to low cell counts.

To describe participants, the pre-intervention questionnaire also included participant practice and education characteristics. To determine intervention engagement, attendance at each session was tracked.

### Outcomes

The primary outcomes were the proportion of included health records with a completed reactive balance measure pre- (objective i), during- (objective ii) and post-intervention (objective iii). Secondary outcomes included differences in reactive balance measure use during- and post-intervention, differences in reactive balance measure use across sites, differences in reactive balance knowledge and measurement confidence pre- and post-intervention, participant satisfaction with intervention content, delivery, and intervention components, and intent to continue reactive balance measurement.

### Sample size estimates

Sample size decisions were based on the during-intervention outcome of proportion of health records with the reactive balance measure. Decisions were guided by a review of previous implementation research effect sizes [38]. Anticipating a 20% effect size during-intervention, reference calculations indicated that a sample size of 91 health records was required to demonstrate a 20% increase in the proportion of health records with the reactive balance measure completed during-intervention (alpha level = 0.05; 80% power) [39]. This estimate was increased to 100 for each of the three sites (i.e. total during-intervention sample size *n* = 300). Sample size decisions for pre- and post-intervention periods were based on feasibility of data collection considerations within the funded granting period.

### Statistical analysis

To address the primary objectives to determine reactive balance measure use (primary objectives i and ii), descriptive statistics were calculated for pre-, during-, and post-intervention periods. To address the primary objective determining how reactive measure use was sustained (primary objective iii), chi-square tests using predicted proportions for the expected values were used to determine changes in rates of reactive balance measurement during and post-intervention. To address secondary objectives, differences in health record characteristics across sites were compared with Kruskall-Wallis tests for non-parametric continuous data or chi-square tests for categorical data as appropriate, two-way repeated measures ANOVA were used to determine differences in total reactive balance knowledge and confidence by site (3 levels) and time (pre- and post- intervention), and chi-square tests using predicted proportions for the expected values were used to determine differences in satisfaction ratings across sites. Significance level was set at *p* < 0.05. Values expressed are mean ± standard deviation.

## Results

### Pre-intervention baseline reactive balance measure use

Of the 300 records reviewed in the pre-intervention period, 211 included evidence of balance assessment and were eligible. Among these, a standardized reactive balance measure was not documented in any health record. Within one record (0.5%), there was a completed non-standardized reactive balance assessment, and 15 records (7%) mentioned reactive balance in the physiotherapy notes section of the health record.

### Participants

Thirty-three physiotherapists enrolled in the study. Five participants (15%) withdrew during the intervention, either voluntarily (*n* = 2) or due to change in job status (*n* = 3). Twenty-eight participants completed the study (Table 2). Participants had on average 12 ± 2 years of clinical experience, and most participants (*n* = 19, 68%) estimated that in a typical week, at least 80% of their caseload included adults with balance impairment at risk of falls. Participants worked with a diverse range of clinical populations, including neurological (*n* = 11, 39%), orthopedic (*n* = 8, 29%), geriatric (*n* = 3, 11%), and those with multiple or complex conditions in need of general reconditioning (*n* = 5, 18%). Most participants (*n* = 20, 71%) worked in inpatient settings.

**Table 2:** Participant characteristics by site (Mean ± SD or n (%))

Characteristic	Site 1(*n* = 10)	Site 2 (*n* = 7)	Site 3 (*n* = 11)
Female	7 (70%)	6 (86%)	9 (81%)
Years since graduation	9.6 ± 4.7	13.9 ± 7.6	12.9 ± 6.3
Highest degree attained			
Bachelor’s	4 (40%)	4 (57%)	4 (36%)
Entry-Level Master’s	5 (50%)	2 (29%)	7 (64%)
Research Master’s	1 (10%)	1 (14%)	0
Primary area of practice			
Neurological	6 (60%)	5 (71%)	0
Orthopedic	1 (10%)	0	7 (64%)
Geriatric	0	0	4 (36%)
Multiple/ complex conditions	3 (30%)	2 (29%)	0
Clinical program type			
Inpatient	5 (50%	5 (71%)	10 (91%)
Outpatient	1 (10%)	2 (29%)	1 (9%)
Undesignated	4 (40%)	0	0
Estimated proportion of caseload with balance impairment at risk of falls in a typical week			
1–39%	2 (20%)	0	0
40–59%	0	1 (14%)	2 (18%)
60–79%	4 (40%)	0	0
80 + %	4 (40%)	6 (86%)	9 (82%)
Didactic education meeting attendance	10 (100%)	7 (100%)	10 (91%)
Hands-on practice meeting attendance	10 (100%)	7 (100%)	11 (100%)
Check-in discussion meeting attendance (/5)			
5	4 (40%)	2 (29%)	3 (27%)
4	3 (30%)	4 (57%)	3 (27%)
3	2 (20%)	1 (14%)	3 (27%)
2	0	0	2 (18%)
1	1 (10%)	0	0

### Intervention delivery

All three local champions at each site were actively involved throughout the intervention. All champions were in attendance and co-facilitated all seven meetings at each site and responded to all participant inquiries and brokered questions to the research team. Local champion contributions were fluid and responsive to participant needs. All seven group meetings were held as planned at each site (total *n* = 21 group meeting sessions). Average attendance at the group meetings was six out of seven meetings. Seven participants (25%) attended all meetings. Five participants (18%) attended four or fewer meetings. Attendance details are reported in Table 2. The reactive balance measure administration form was co-developed and revised over multiple drafts and included content and layout feedback from participants. Health record modification processes were tailored to each site and practice setting as needed. Reactive balance measure administration forms were stored in the additional notes sections of the health records.

### Protocol modifications

Initial published intervention components [28] included using regular health record audits as an additional feedback strategy to participants. However, this component was not delivered due to feasibility challenges extracting participant-linked data in real-time during the intervention. There were also unanticipated challenges and delays in linking the reactive balance measure administration form to the health record and participant caseload at the start of the intervention for all sites. Participants were encouraged to use the measure during this period, however the period of health record eligibility was reduced to the final 8–9 months of the 12-month intervention.

### Reactive balance measure use during- and post- intervention

**Fig. 2:**
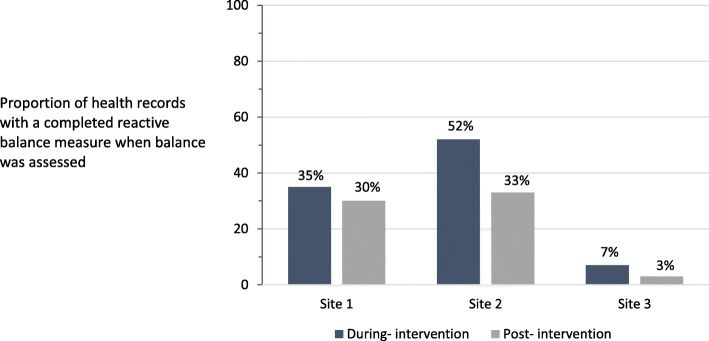
During- and post-intervention reactive balance measure use by site. Site differences were significant during- intervention (*χ*^2^ = 48.0, *p* < 0.0001) and post-intervention (*χ*^2^ = 8.8, *p* < 0.12)

During-intervention, the reactive balance measure was documented in 31% of eligible health records (*n* = 94). Post-intervention, the reactive balance measure was documented in 19% of eligible health records (*n* = 18). The proportion of eligible health records with a completed reactive balance measure was significantly lower post-intervention compared to during-intervention (X^2^ = 4.34, *p* = 0.037). In both during- and post-intervention periods, there was a significant difference in the proportion of eligible health records with a completed reactive balance measure across sites that ranged from 7 to 52% of records during-intervention and 3–35% of records post-intervention (all *p* < = 0.012, Fig. 2). Health record characteristics are reported in Additional file 4.

### Reactive balance knowledge and measurement confidence pre- and post- intervention

Reactive balance knowledge significantly improved across all three sites (pre: 65.3 ± 13.5%; post: 74.8 ± 12.4%; time main effect (F (1, 25) = 9.1, *p* < 0.01). Self-reported confidence for reactive balance measurement also significantly improved across all three sites (pre: 54.3 ± 20.9%; post: 76.2 ± 14.9%; time main effect (F (1, 25) = 20.1, *p* < 0.001). There were no significant interactions or differences in reactive balance knowledge or confidence for reactive balance measurement across sites (all *p* > 0.05).

### Intervention satisfaction, utility and intent to use reactive balance measure

Most participants reported positive satisfaction with the intervention content (*n* = 20, 71%) and delivery (*n* = 19, 68%). With respect to utility of intervention components, the hands-on practice meeting received the most positive ratings (*n* = 27, 96%). Eighty-two percent (*n* = 23) of participants positively rated the utility of the initial didactic education meeting, 71% (*n* = 20) positively rated utility of the health record modifications, 61% (*n* = 17) positively rated the utility of the bi-monthly check-in discussion meetings, and 46% (*n* = 13) positively rated the utility of local champions. Forty three percent of participants (*n* = 12) indicated that they intended to use the reactive balance measure on completion of the study. There were no significant differences in any ratings across sites (all *p* > 0.05).

## Discussion

The primary goal of this research was to improve physiotherapist use of reactive balance measurement in rehabilitation settings through a theory-based, multi-component intervention targeted to address established factors influencing reactive balance measurement. Key findings were (i) evidence of positive effects in reactive balance measurement use, knowledge, and confidence during- and post-intervention; (ii) partial sustainment of reactive balance measure use on completion of the intervention; and (iii) significant differences in reactive balance measure use across sites. There is limited published data on which to provide contextual reference points for interpreting the findings. A 2017 systematic review on rehabilitation measurement interventions showed that 9 of 10 studies measuring rates of standardized outcome measure use demonstrated improvements [40], as did this study. Reported percentage improvement rates ranged between 15 and 35%, though pre-intervention usage rates started as high as 61% [40]. The complete pre-intervention absence of the measurement behavior is a unique aspect of this study. With regards to a contextual reference for reactive balance measurement use, there is one published study [41] describing implementation of a different reactive balance assessment protocol with an inpatient sub-acute stroke population through a clinic partnering researchers and physiotherapists. They reported that 42% of inpatient sub-acute stroke admissions received a reactive balance assessment in a 12 –month period. This is higher than reactive measure use observed in the present study, although use of a difference reactive balance assessment protocol and research staff involvement in data collection may account for some of the differences.

This quasi-experimental study makes important advances to both implementation science and to the application of evidence in rehabilitation practice. With regards to the contribution to implementation science, this study offers important insights about the potential active ingredients of the intervention and ongoing sustainability issues. This study is among the first to use a determinant framework, and more specifically, the TDF, to directly link barriers and facilitators to rehabilitation intervention components, strategies and outcomes. The hands-on practice meeting – the most positively rated component by participants – suggests that rehearsal of relevant skills, feedback, problem solving, decision making, goal setting, and modeling/demonstration of behaviors by others [28, 32] may be related to intervention effects. However, this cannot be confirmed in the absence of an appropriate comparator for this intervention. The significant decrease in reactive balance measurement during- to post-intervention highlights the ongoing issue of the time and resources required to fully integrate new practices into routine care. Despite specific attention paid to sustainability considerations in the design of the study, active engagement of participants during-intervention was related to reactive measure use. As long-term follow-up may be outside the scope of a research study, partnerships between researchers and users may facilitate sustainability planning for continued active engagement in the practice behavior.

The multi-site research design demonstrates that even a highly-targeted intervention can have a range of effects, and the observed range of reactive balance measure use across sites speaks to the study’s contribution to understanding the application of evidence in rehabilitation practice. For example, differing site population characteristics may be a contributing factor influencing reactive balance measurement. However, the direction of the relationship between patient factors and reactive balance measurement is not clear. Our previous research has shown that primary area of practice is a significant predictor of self-reported reactive balance assessment practice [21]. While others have reported that physiotherapists identify patient factors as driving their clinical reasoning for balance measurement [42], future studies may investigate the potential for clinical practice area cultural trends within a profession. Organizational culture, the shared beliefs, attitudes, values, and behavioural norms of an institution [43], may also have contributed to varied findings across sites. For example, given strong links between health care organization accreditation policies and the dedicated focus on safety and “preventable falls” in Canada, there may be some influence on clinician perceptions about risk and willingness to take risk with patients. Continued research to understand the intersections of organizational culture, clinical norms, patient characteristics, and implementation intervention characteristics, both generally and specific to reactive balance measurement, are warranted.

### Limitations

The study’s quasi-experimental design limits the extent of causational conclusions that can be drawn due to persisting threats to internal validity. Future studies should explore alternative study designs (e.g. stepped wedge [44]) to address this limitation. The inability to directly compare during- and post-intervention reactive measure use with pre-intervention use due to differences in data sampling is also a limitation. The decision to conduct bi-monthly check-in discussion meetings was not based on published evidence and warrants continued research. The knowledge questionnaire did not undergo a full psychometric evaluation and needs to be validated and further developed to fully understand the clinical relevance of the changes in scores. Recent advancements in standardized approaches for measuring clinical practice and professional development (e.g. [45]) could strengthen this component of data collection. The post-intervention data collection was smaller than the during-intervention and the reduced precision of estimating documented use at that stage is a limitation. Finally, it is acknowledged that the intervention protocol was modified by not delivering health record audit and feedback as planned. While such audit and feedback techniques have demonstrated effectiveness [46], the TDF domain targeted through the monitoring technique (skills) was addressed by other techniques used in the intervention (e.g., rehearsal of relevant skills, problem solving, decision making, goal setting).

## Conclusions

While reactive balance measure use was greater during the theory-based multi-component intervention relative to pre-intervention baseline period, effects varied across rehabilitation settings and use was only partially sustained. Participant satisfaction ratings indicated the hands-on practice meetings were perceived as most useful for supporting implementation of reactive measurement. Forthcoming research will explore how the intervention influenced clinical reasoning and implementation fidelity, and the patient factors that were associated with reactive balance measure use. Future work may explore impact of a theory-based intervention, long-term sustainability of the intervention, comparative effects, and efficacy in various rehabilitation sub-settings to further elucidate the potential for impact.

## Additional files

Additional file 1: Reactive balance measure administration form. (DOC 58 kb)

Additional file 2: Meeting agendas. (DOC 32 kb)

Additional file 3: Questionnaire. (DOC 85 kb)

Additional file 4: Health record characteristics. (DOC 42 kb)

## Abbreviations

FTE: Full-Time Equivalent
TDF: Theoretical Domains Framework
