# Supplementary material for: A theory-based multi-component intervention to increase reactive balance measurement by physiotherapists in three rehabilitation hospitals: an uncontrolled single group study
Source: BMC Health Serv Res. 2018 Sep 19;18:724. doi: 10.1186/s12913-018-3533-8 (PMC6146937; doi:10.1186/s12913-018-3533-8)
Supplement: Supplementary file 1 — Reactive balance measure administration form. (DOC 58 kb) [file 12913_2018_3533_MOESM1_ESM.doc]

| **REACTIVE POSTURAL RESPONSES SECTION OF**  **THE BALANCE EVALUATION SYSTEMS TEST (BESTEST) TRACKING FORM**  ****Please retain in paper chart**** |  |
| --- | --- |

|  | | **Date:**  **dd/mm/yy** | **__/__/__** | **__/__/__** | **__/__/__** | **__/__/__** |
| --- | --- | --- | --- | --- | --- | --- |
| **Is the BESTest an appropriate measure for this patient?** | | | **□ No**;  □ cannot stand 30s  □ other: __________  **□ Yes**, see scoring below | **□ No**;  □ cannot stand 30s  □ other: __________  **□ Yes**, see scoring below | **□ No**;  □ cannot stand 30s  □ other: __________  **□ Yes**, see scoring below | **□ No**;  □ cannot stand 30s  □ other: _________  **□ Yes**, see scoring below |
| **Examiner instructions:** | **Patient Instructions** | **Scoring:** |  |  |  |  |
| **In place response- *forward:***  Stand in front of the patient, place one hand on each shoulder and lightly push the patient backward until their anterior ankle muscles contract, (and toes just start to extend) then suddenly release. Do not allow any leaning by patient. Score only the best of 2 responses if the patient is unprepared or you pushed too hard. | For the next few tests, I’m going to push against you to test your balance reaction. Stand in your normal posture with your feet shoulder-width apart, arms at your sides. Do not allow my hands to push you backward When I let go, keep your balance without taking a step. | **(3)** Recovers stability with ankles, no added arms or hips motion  **(2)** Recovers stability with arm or hip motion  **(1)** Takes a step to recover stability  **(0)** Would fall if not caught OR requires assist OR will not attempt | 3 □  2 □  1 □  0 □ | 3 □  2 □  1 □  0 □ | 3 □  2 □  1 □  0 □ | 3 □  2 □  1 □  0 □ |
| **In place response- *backward*:**  Stand behind patient, place one hand on each scapula and isometrically hold against patient’s backward push, until heels are about to be lifted, not allowing trunk motion. Suddenly release. Do not allow any leaning by patient. Score only the best of 2 responses if patient is unprepared, or you pushed too hard. | Stand with your feet shoulder width apart, arms at your sides. Do not allow my hands to push you forward. When I let go, try to keep your balance without taking a step. | **(3)** Recovers stability at ankles, no added arm/ hip motion  **(2)** Recovers stability with some arm or hip motion  **(1)** Takes a step to recover stability  **(0)** Would fall if not caught OR requires assist OR will not attempt | 3 □  2 □  1 □  0 □ | 3 □  2 □  1 □  0 □ | 3 □  2 □  1 □  0 □ | 3 □  2 □  1 □  0 □ |

| **Examiner instructions:** | **Patient Instructions** | **Scoring: Date:** | | **__/__/__** | | | __/__/__ | | | **__/__/__** | | | **__/__/__** | |
| --- | --- | --- | --- | --- | --- | --- | --- | --- | --- | --- | --- | --- | --- | --- |
| **Compensatory stepping correction- forward:**  Stand in front to the side of patient with one hand on each shoulder and ask them to push forward. (Make sure there is room for them to step forward). Requires them to lean until their shoulders and hips are in front of their toes. Suddenly relate your push when the subject is in place and providing constant pressure to a level just before the hells lift off. The test must elicit at step. NOTE: be prepared to catch patient. | Stand with your feet should width apart, arms at your sides. Lean forward against my hands beyond your forward limits. When I let go, do whatever is necessary, including taking a step, to avoid a fall. | **(3)** Recovers independently a single, large step (second realignment step is allowed)  **(2)** More than one step used to recover equilibrium, but recovers stability independently OR 1 step with imbalance.  **(1)** Takes multiple steps to recover equilibrium, or needs minimum assistance to prevent a fall  **(0)** No step, OR would fall if not caught, OR falls spontaneously | | 3 □  2 □  1 □  0 □ | | | 3 □  2 □  1 □  0 □ | | | 3 □  2 □  1 □  0 □ | | | 3 □  2 □  1 □  0 □ | |
| **Compensatory stepping correction- backward:**  Stand in back to the side of the patient with one hand on each scapula and ask them to push backward. (Make sure there is room for them to step backward.) Require them to lean until their shoulders and hips are in back of their heels. Release your push when the subject is in place, and providing constant pressure to a level just before the heels lift off. Test must elicit at step. NOTE: Be prepared to catch patient. | Stand with your feet shoulder width apart, arms down at your sides. Lean backward against my hands beyond your backward limits. When I let go, do whatever is necessary, including taking a step, to avoid a fall. | **(3)** Recovers independently a single, large step  **(2)** Takes 2-3 steps and recovers independently OR 1 step with imbalance.  **(1)** Takes >3 steps to recover equilibrium, OR needs minimum assistance  **(0)** No step, OR would fall if not caught, OR falls spontaneously | | 3 □  2 □  1 □  0 □ | | | 3 □  2 □  1 □  0 □ | | | 3 □  2 □  1 □  0 □ | | | 3 □  2 □  1 □  0 □ | |
| **Compensatory stepping correction- lateral:**  Stand in behind the patient, place one hand on either the right (or left) side of the pelvis, and get them to lean their whole body into your hand. Require them to lean until the midline of pelvis is over the right (or left) foot and then suddenly release your hold. NOTE: Be prepared to catch patient. | Stand with your feet together, arms down at your sides. Lean into my hand beyond your sideways limit. When I let go, step if you need to, to avoid a fall. | **Left/Right side:**  **(3)** Recovers independently with 1 step of normal length/ width (crossover or lateral OK)  **(2)** Several steps used, but recovers independently  **(1)** Steps, but needs to be assisted to prevent a fall  **(0)** Falls, or cannot step | | **L**  3 □  2 □  1 □  0 □ | **R**  3 □  2 □  1 □  0 □ | | **L**  3 □  2 □  1 □  0 □ | **R**  3 □  2 □  1 □  0 □ | | **L**  3□  2□  1□  0□ | **R**  3□  2□  1□  0□ | | **L**  3□  2□  1□  0□ | **R**  3□  2□  1□  0□ |
| _______________________ ________________________ ___________________ Total Score /18  Physiotherapist (Print Name) Physiotherapist’s Signature Date: (DD/MMM/YYYY) Therapist Initials | | |  | | |  | | |  | | |  | | |
|  | | |  | | |  | | |  | | |
