# Supplementary material for: A theory-based multi-component intervention to increase reactive balance measurement by physiotherapists in three rehabilitation hospitals: an uncontrolled single group study
Source: BMC Health Serv Res. 2018 Sep 19;18:724. doi: 10.1186/s12913-018-3533-8 (PMC6146937; doi:10.1186/s12913-018-3533-8)
Supplement: Supplementary file 2 — Meeting agendas. (DOC 32 kb) [file 12913_2018_3533_MOESM2_ESM.doc]

Supplemental file 1: Group meeting agendas

Didactic Education Session (60 min)

1. Welcome and introductions
2. Objective and outline of session
   1. Introduce the REACT study
   2. Review existing data on reactive balance and relationship to falls
   3. Review measurement options for reactive balance and what does/ doesn’t evaluate it
   4. Introduce the measure at the focus of the REACT study
3. REACT study—background and what will happen
   1. Objectives
   2. Study team/ sites
   3. Timelines
   4. Intervention components
   5. Data collection points/ commitments
   6. Questions about the study?
4. What is reactive balance?
   1. One of many important elements of postural control
   2. Types of reactive balance- internal vs external, fixed- support, change in support reactions
5. Feature of reactive balance related to falls
6. Measuring reactive balance
   1. Why measure?
   2. What measures don’t evaluate reactive balance and why
   3. What measures do evaluate reactive balance
7. The REACT measure: the Balance Evaluation Systems test
   1. What is it? How was it developed?
   2. Reactive postural response section
      1. Description
      2. Psychometric data
      3. Populations used in
      4. Demonstration/ videos
      5. Scoring
      6. Introduce chart sheet
8. Summary and take home messages
9. Next session preview
10. Questions

Hands-on Practice Session (60 min)

1. Session #1 reflection
2. BESTest postural responses section
   1. In place- forward and backward (2 items)
      1. Review examiner instructions (discuss and watch video)
      2. Review patient instructions (discuss and watch video)
      3. Review scoring criteria (discuss and watch video for each scoring level)
      4. Practice administration technique on each other and discuss questions, concerns as needed
   2. Forward step
      1. Review examiner instructions (discuss and watch video)
      2. Review patient instructions (discuss and watch video)
      3. Review scoring criteria (discuss and watch video for each scoring level)
      4. Practice administration technique on each other and discuss questions, concerns as needed
   3. Backward step
      1. Review examiner instructions (discuss and watch video)
      2. Review patient instructions (discuss and watch video)
      3. Review scoring criteria (discuss and watch video for each scoring level)
      4. Practice administration technique on each other and discuss questions, concerns as needed
   4. Lateral step (2 items)
      1. Review examiner instructions (discuss and watch video)
      2. Review patient instructions (discuss and watch video)
      3. Review scoring criteria (discuss and watch video for each scoring level)
      4. Practice administration technique on each other and discuss questions, concerns as needed
3. Review administration form
4. Questions, concerns?

Check-in Session (Bi-monthly x 5)

1. Welcome and study logistic update from researchers
2. Participant discussion and thoughts to date
   1. How much have you used test?
   2. Experiences?
      1. Deciding whom to use with
      2. Administering perturbations
      3. Evaluating performance (scoring criteria)
      4. Utility of form
   3. Specific challenges and successes so far?
   4. Questions/ issues that have arisen that you’re unsure how to deal with
   5. Process of using BESTest form and leaving in folder- How is that working?
   6. Other issues/ questions/ concerns?
