# Supplementary material for: A theory-based multi-component intervention to increase reactive balance measurement by physiotherapists in three rehabilitation hospitals: an uncontrolled single group study
Source: BMC Health Serv Res. 2018 Sep 19;18:724. doi: 10.1186/s12913-018-3533-8 (PMC6146937; doi:10.1186/s12913-018-3533-8)
Supplement: Supplementary file 3 — Questionnaire. (DOC 85 kb) [file 12913_2018_3533_MOESM3_ESM.doc]

***Thank you for participating in the REACT study! We sincerely appreciate your time and contribution, and are interested in understanding if and how the program is useful for your practice. This questionnaire explores your knowledge and confidence about reactive balance measurement, and will be completed at the beginning and end of the study.***

**Participant ID: ____________________**

**Questionnaire time point:**

- Pre-intervention
- Post-intervention

**Date of completion (YY/ MM/ DD):** _________________

**This section asks about your understanding of reactive balance and ways of evaluating it.** *Please answer the following questions*.

Which of the following are possible reactive postural control strategies? (Check all that apply)

- Muscle contractions about the ankle joint in a stationary position
- Muscle contractions about the hip joint in a stationary position
- Stepping
- Upper limb grasping of a stabilizing support

Which of the following reactive postural control response patterns are associated with increased risk of falls in older adults? (Check all that apply)

- Slower lower limb muscle responses
- Multiple steps
- Upper limb grasping of a stabilizing support
- Smaller lower limb muscle responses

Which of the following tasks evaluate reactive postural control? (Check all that apply)

- Externally-induced perturbation
- One-legged stance
- Walking
- Standing with eyes closed
- Standing on foam
- Reaching to pick an object off of floor

Which of the following measures include a task that evaluates reactive postural control? (Check all that apply)

- Berg Balance Scale
- Timed Up and Go test
- Single Leg Stance test
- Chedoke-McMaster Stroke Assessment scale
- Balance Evaluation Systems test (BESTest)
- Community Balance and Mobility scale
- Performance Oriented Mobility Assessment

**This section asks about your confidence in assessing and measuring reactive balance in your caseload. *Please answer the following questions*.**

| How confident are you in your ability to: | Certain Cannot do | |  |  | Moderately  Certain  Can do | | |  |  | Certain Can Do | |
| --- | --- | --- | --- | --- | --- | --- | --- | --- | --- | --- | --- |
|  | 0% | 10% | 20% | 30% | 40% | 50% | 60% | 70% | 80% | 90% | 100% |
| Identify appropriate patients for reactive balance assessment? |  |  |  |  |  |  |  |  |  |  |  |
| Select an appropriate method of assessing reactive balance? |  |  |  |  |  |  |  |  |  |  |  |
| Safely administer a validated measure of reactive balance? |  |  |  |  |  |  |  |  |  |  |  |
| Interpreting patients’ performance on validated measures of reactive balance? |  |  |  |  |  |  |  |  |  |  |  |

**This section asks about you and your practice.** *Please answer the following questions*.

What percentage of your caseload includes:

Adults (18-64 years)?

| 0% | 1-19% | 20-39% | 40-59% | 60-79% | ≥80% |
| --- | --- | --- | --- | --- | --- |

Older adults (≥65 years)?

| 0% | 1-19% | 20-39% | 40-59% | 60-79% | ≥80% |  |  |  |  |
| --- | --- | --- | --- | --- | --- | --- | --- | --- | --- |

In a typical week, what percentage of your caseload includes adults with balance impairments who are at risk of falls?

| 0% | 1-19% | 20-39% | 40-59% | 60-79% | ≥80% |
| --- | --- | --- | --- | --- | --- |

In a typical week, what percentage of your caseload includes adults with balance impairments who are at risk of falls and are able to stand independently for 30 seconds?

| 0% | 1-19% | 20-39% | 40-59% | 60-79% | ≥80% |
| --- | --- | --- | --- | --- | --- |

In a typical week, which best describes your primary area of practice treating adults with balance impairments at risk of falls? *(Choose ONE only)*

| Neurology | Orthopedics | Cardiorespiratory |
| --- | --- | --- |
| Sports Injuries | Geriatric | Vestibular |
| Multiple/ complex chronic conditions | Other (specify): ____________________________________ | |

How long have you been assessing and treating adults with balance impairments at risk of falls?

| _____ Years | _____ Months |
| --- | --- |

How old are you?

| ≤30 years | 31-40 years | 41-50 years | 51-60 years | ≥60 years |
| --- | --- | --- | --- | --- |

What is your gender?

| Female | Male |
| --- | --- |

What is your entry-to-practice degree for Physical Therapy?

| Diploma | Bachelor’s | Entry-level Master’s | Other (specify): ______________________________ |
| --- | --- | --- | --- |

What year did you graduate from your Physical Therapy program? _______________

What is your highest degree attained?

| Diploma | Bachelor’s | Entry-level Master’s |
| --- | --- | --- |
| Applied or Research Master’s | Doctoral | Other (specify):__________________ |

**This section asks about your *personal perspective(s)* related to the REACT study.** *Please answer the following questions*.

Please rate your overall satisfaction with the content of the REACT intervention.

| Strongly Negative | Negative | Slightly Negative | Neutral Opinion | Slightly Positive | Positive | Strongly Positive |
| --- | --- | --- | --- | --- | --- | --- |

Please rate your overall satisfaction with the delivery of the REACT intervention.

| Strongly Negative | Negative | Slightly Negative | Neutral Opinion | Slightly Positive | Positive | Strongly Positive |
| --- | --- | --- | --- | --- | --- | --- |

Please rate the *utility* of the *initial didactic lecture*.

| Very useless | Useless | Slightly useless | Neutral | Slightly useful | Useful | Very Useful |
| --- | --- | --- | --- | --- | --- | --- |

Please rate the *utility* of the *initial hands-on seminar*.

| Very unlikely | Unlikely | Slightly unlikely | Neutral | Slightly likely | Likely | Very likely |
| --- | --- | --- | --- | --- | --- | --- |

Please rate the *utility* of the *bi-monthly check-ins*.

| Very unlikely | Unlikely | Slightly unlikely | Neutral | Slightly likely | Likely | Very likely |
| --- | --- | --- | --- | --- | --- | --- |

Please rate the *utility* of the *local site champion role*.

| Very unlikely | Unlikely | Slightly unlikely | Neutral | Slightly likely | Likely | Very likely |
| --- | --- | --- | --- | --- | --- | --- |

Please rate the *utility* of the *chart modifications* that were made to facilitate the use of the BESTest.

| Very unlikely | Unlikely | Slightly unlikely | Neutral | Slightly likely | Likely | Very likely |
| --- | --- | --- | --- | --- | --- | --- |

How likely are you to continue to use the BESTest Reactive Postural Responses test after the REACT study is over?

| Very unlikely | Unlikely | Slightly unlikely | Neutral | Slightly likely | Likely | Very likely |
| --- | --- | --- | --- | --- | --- | --- |
