# Supplementary material for: A theory-based multi-component intervention to increase reactive balance measurement by physiotherapists in three rehabilitation hospitals: an uncontrolled single group study
Source: BMC Health Serv Res. 2018 Sep 19;18:724. doi: 10.1186/s12913-018-3533-8 (PMC6146937; doi:10.1186/s12913-018-3533-8)
Supplement: Supplementary file 4 — Health record characteristics. (DOC 42 kb) [file 12913_2018_3533_MOESM4_ESM.doc]

**Supplemental Table**. Health record characteristics by site

| **Site** | **1** | | **2** | | **3** | | **X^2^ (p)** | |
| --- | --- | --- | --- | --- | --- | --- | --- | --- |
| **Time Period** | **During** | **Post** | **During** | **Post** | **During** | **Post** | **During** | **Post** |
| % Male | 53 | 50 | 52 | 55 | 41 | 10 | 3.5 (0.17) | 1.4 (0.49) |
| Median age, years | 71 | 75 | 68.5 | 67 | 76.5 | 80 | 15. 2 (0.0005) | 5.8 (0.055) |
| Median length of stay, days | 23 | 21.5 | 30 | 42 | 32 | 25.5 | 5.9 (0.053) | 14.6 (0.0007) |
| % with primary diagnosis |  |  |  |  |  |  | 120.0 (0.0001) | 24.2 (0.0021) |
| Neurological condition | 68 | 46.7 | 79 | 73.3 | 9 | 28 |  |  |
| Orthopedic condition | 8 | 13.3 | 2 | 10 | 11 | 17.2 |  |  |
| Debility | 12 | 3.3 | 3 | 6.7 | 30 | 31 |  |  |
| Other | 12 | 36.7 | 15 | 6.7 | 49 | 20.7 |  |  |
| Missing | 0 |  | 1 | 3.3 | 1 | 3.3 |  |  |
| % inpatient | 75 | 86.7 | 94 | 25 | 75 | 72.4 | 14.3 (0.0008) | 25.4 (0.0001) |
| % with completed reactive balance measure | 35 | 20 | 52 | 33 | 7 | 3 | 48.0 (0.0001) | 8.8 (0.012) |
| Median reactive balance measure score, /18 | 15 | 14 | 13.5 | 15 | 10.5 | 5 | 2.1 (0.35) | 3.04 (0.22) |
| % with other completed balance measures |  |  |  |  |  |  |  |  |
| Berg Balance Scale | 95 | 93 | 98 | 100 | 96 | 100 | - | 4.1 (0.13) |
| Community Balance and Mobility Scale | 27 | 6.7 | 5 | 6.7 | 5 | 0 | 29.8 (0.0001) | - |
| Timed Up-and-Go Test | 4 | 3.3 | 0 | 0 | 27 | 33.3 | 45.8 (< 0.0001) | - |
| Median Berg Balance Scale score, /56 | 45 | 45 | 49 | 45 | 43 | 43.2 | 18.6 (0.0001) | 0.085 (0.96) |
